# Increased 5-hydroxymethylcytosine and decreased 5-methylcytosine are indicators of global epigenetic dysregulation in diffuse intrinsic pontine glioma

**DOI:** 10.1186/2051-5960-2-59

**Published:** 2014-06-03

**Authors:** Sama Ahsan, Eric H Raabe, Michael C Haffner, Ajay Vaghasia, Katherine E Warren, Martha Quezado, Leomar Y Ballester, Javad Nazarian, Charles G Eberhart, Fausto J Rodriguez

**Affiliations:** Johns Hopkins Division of Pediatric Oncology, Baltimore, 21218 MD USA; Sidney Kimmel Comprehensive Cancer Center, Johns Hopkins Hospital, 1800 Orleans St, 21287 Baltimore, MD USA; National Institutes of Health, National Cancer Institute, Pediatric Oncology Branch, Bethesda, MD USA; National Cancer Institute, Laboratory of Pathology, National Institutes of Health, Bethesda, MD USA; Center for Genetic Medicine, Children’s National Medical Center, 111 Michigan Ave NW, 20010 Washington, DC USA; Department of Integrative Systems Biology, George Washington University School of Medicine and Health Sciences, Washington, 20010 DC USA; Division of Neuropathology, Johns Hopkins University, Baltimore, 21218 MD USA

**Keywords:** DIPG, H3F3A, 5-hydroxymethylcytosine, 5-methylcytosine, H3K27 trimethylation, H3K9 trimethylation

## Abstract

**Introduction:**

Diffuse intrinsic pontine glioma (DIPG) is a malignant pediatric brain tumor associated with dismal outcome. Recent high-throughput molecular studies have shown a high frequency of mutations in histone-encoding genes (*H3F3A* and *HIST1B*) and distinctive epigenetic alterations in these tumors. Epigenetic alterations described in DIPG include global DNA hypomethylation. In addition to the generally repressive methylcytosine DNA alteration, 5-hydroxymethylation of cytosine (5hmC) is recognized as an epigenetic mark associated with active chromatin. We hypothesized that in addition to alterations in DNA methylation, that there would be changes in 5hmC. To test this hypothesis, we performed immunohistochemical studies to compare epigenetic alterations in DIPG to extrapontine adult and pediatric glioblastoma (GBM) and normal brain. A total of 124 tumors were scored for histone 3 lysine 27 trimethylation (H3K27me3) and histone 3 lysine 9 trimethylation (H3K9me3) and 104 for 5hmC and 5-methylcytosine (5mC). An H-score was derived by multiplying intensity (0–2) by percentage of positive tumor nuclei (0-100%).

**Results:**

We identified decreased H3K27me3 in the DIPG cohort compared to pediatric GBM (p < 0.01), adult GBM (p < 0.0001) and normal brain (p < 0.0001). H3K9me3 was not significantly different between tumor types. Global DNA methylation as measured by 5mC levels were significantly lower in DIPG compared to pediatric GBM (p < 0.001), adult GBM (p < 0.01), and normal brain (p < 0.01). Conversely, 5hmC levels were significantly higher in DIPG compared to pediatric GBM (p < 0.0001) and adult GBM (p < 0.0001). Additionally, in an independent set of DIPG tumor samples, *TET1* and *TET3* mRNAs were found to be overexpressed relative to matched normal brain.

**Conclusions:**

Our findings extend the immunohistochemical study of epigenetic alterations in archival tissue to DIPG specimens. Low H3K27me3, decreased 5mC and increased 5hmC are characteristic of DIPG in comparison with extrapontine GBM. In DIPG, the relative imbalance of 5mC compared to 5hmC may represent an opportunity for therapeutic intervention.

**Electronic supplementary material:**

The online version of this article (doi:10.1186/2051-5960-2-59) contains supplementary material, which is available to authorized users.

## Introduction

Tumors of the central nervous system are the second most common malignancy in pediatric patients. Diffuse intrinsic pontine gliomas (DIPGs) comprise approximately 10% of pediatric brain tumors and are universally fatal [1]. Due to their location, DIPGs have rarely been biopsied at diagnosis outside of recent clinical trials. No improvements in DIPG outcome have been noted in the past 20 years, perhaps due to the paucity of patient-derived samples and lack of molecular insights leading to novel treatments. Encouragingly, recent molecular and proteomic analyses of autopsy specimens have identified key genetic alterations in DIPG, including amplifications in genes encoding receptor tyrosine kinases (*PDGFRA*, *MET*) [2], *PDGFRA* mutations [3], as well as distinct DIPG subgroups based on Hedgehog (SHH) and MYCN pathway activation [4]. Mutations in the *ACVR1* gene, encoding the transforming growth factor-beta (TGF-beta) superfamily member activin, have been reported in approximately 20% of DIPGs [5, 6].

Epigenetic research has added to our understanding of how chromatin remodeling by methylation and acetylation of histones affects gene expression in tumors [7]. Among brain tumors, glioblastomas (GBMs) can be subdivided into 6 groups based on DNA methylation patterns. DIPGs are classified within one group that has relatively hypomethylated DNA and are associated with mutations in genes encoding for histone proteins [8–11]. Approximately 60% of DIPGs have a mutation in the *H3F3A* gene which encodes the variant histone 3.3 proteins [11], which is also associated with a worse prognosis [12]. Less frequently, DIPGs have mutations in *HIST1H3B*, encoding for histone H3.1, while other extrapontine pediatric high-grade gliomas may have alternative mutations in *H3F3A* (G34R or G34V) [13, 8, 9]. As a result of the common missense mutation (*H3F3A*), lysine at position 27 (H3K27) is changed to methionine (K27M) in the amino terminal tail of histone 3.3 [10]. Post-translational modification of this critical histone regulates gene expression, DNA repair, and maintenance of centromeres/telomeres [8]. Methylation of the H3K27 is mediated by polycomb repressive complex 2 (PRC2), which binds to polycomb group (PcG) target genes and induces the PRC2 component enhancer of zeste homologue 2 (EZH2) methyltransferase to methylate H3K27. Histone methylation creates a repressed state by inhibiting transcription, altering chromatin compaction, and affecting recruitment of DNA methyltransferases (DNMTs) [8, 14]. The H3K27M mutant histone inhibits PRC2 by interacting with EZH2 and suppressing its function [13]. Therefore, as a result of the K27M mutation, histone methylation is decreased at this site [8, 9, 13, 10]. H3.3 is primarily found at transcription sites and telomeres, and the H3K27M mutant H3.3 is associated with transcriptionally active (open) chromatin [8]. Inhibition of histone K27 tri-methylation (H3K27me3) leads to global activation of transcription [8, 13].

One of the most studied epigenetic alterations is methylation of DNA. Mammalian DNA methylation occurs primarily at the 5-position of cytosines (5mC) in CpG dinucleotides. Methylated cytosines at gene promoters with high GC content (CpG islands) are usually associated with transcriptional silencing. Loss of 5mC leads to a redistribution of PRC2 complexes which suggests that 5mC could affect interactions between PRC2 and chromatin [15]. Aberrant recruitment of PRC2 to DNA not usually associated with H3K27me3 may shift PRC2 away from original targets and again promote an active transcriptional state. H3K27me3 is not uniformly increased in these hypomethylated DNA regions, which provides evidence that DNA methylation is one of many epigenetic factors affecting PRC2 function [15, 10]. A hypomethylated genome, in addition to the *H3F3A* mutation, likely accentuates the transcriptionally active state of DIPGs by disrupting histone methylation at H3K27me3.

The methylation of cytosine in CpG islands is a modification produced by DNMTs. Reversal of 5mC methylation is accomplished in a multistep enzymatic process using Ten Eleven Translocation (TET) enzymes, thymine DNA glycosylase (TDG), and base excision repair (BER) [16, 17]. TET enzymes can convert 5mC in a reaction dependent on alpha-ketoglutarate (α-KG) to 5-hydroxymethylcytosine (5hmC) [16, 18]. 5hmC can then either be further processed by TDG and BER or persist as 5hmC in mammalian genomes [19]. Decreased 5hmC levels have been described in a variety of cancers [20], as well as in high-grade gliomas [21]. 5hmC is often associated with the gene bodies of actively transcribed genes and is considered an epigenetic mark in its own right [17].

These studies raise the possibility that unregulated loss of H3K27me3 through H3K27M mutation and elevated 5hmC could shift normal development into a pathologic state. The association between loss of H3K27me3 and elevated 5hmC in neural development also suggests a regulatory cross talk between these two pathways. Histone 3 lysine 9 methylation (H3K9me3) is another important histone methylation mark implicated in the development of gliomas. Methylation at this site affects global DNA methylation, chromatin compaction, and transcription [22].

In this study we compared epigenetic alterations between DIPG and GBM with respect to patient age and tumor geographical location using archival formalin-fixed paraffin-embedded material to gain a better understanding of epigenetic alterations specific to DIPG.

## Materials and methods

### Ethics statement

Human brain tumor samples and normal control tissue were obtained at biopsy or autopsy at Johns Hopkins Hospital Department of Pathology, Children’s National Medical Center and National Institutes of Health Center for Cancer Research after Institutional Review Board approval or exemption. The research ethics committee waived the requirement for informed consent for retrospective samples and no informed consent was obtained. The patient data was de-identified prior to inclusion in this study.

### Human tissue microarray

DIPG tumor samples were obtained from tissue microarrays created at the National Institutes of Health from rapid autopsy tissue for a total of 24 patients (3 to 15 years of age, with a median age of 7) with each patient having 1–3 representative cores on the array and scored. Tumor samples varied in size with a maximum width of 0.4 cm and maximum length of 1.8 cm. Clinical and pathologic features of this group have been previously published (Additional file 1: Table S1) [1]. The subjects were anonymized as part of the study approval and thus therapeutic data was not collected. Tissue microarrays containing 64 adult GBM (22 to 86 years of age, with median age of 55) and 36 pediatric GBM (less than 1year old to 21 years of age, with median age of 13) were used as a comparison group. Adult and pediatric GBM arrays were created by the Johns Hopkins microarray core facility and have been previously characterized (core diameter 0.6 mm) [23]. Samples with two or more scorable cores were included in our dataset. Cores/samples were excluded from scoring and data analysis if the sample was absent, degraded, or no tumor present. Eight single cores from the GBM arrays and one sample from the DIPG arrays were normal brain and used as controls (i.e. the same normal brain samples from patients were used for comparison between the different IHC stains). Only one normal brain was confirmed to be from the pediatric age group.

### Immunohistochemistry and scoring

The mouse monoclonal anti-Histone H3 (tri methyl K27) antibody ChIP grade (Abcam, Cat# ab6002, Cambridge, MA) was used at 1:1600 dilution. The antibody was incubated overnight at 4°C. For H3K9me3 detection, rabbit polyclonal anti-Histone H3 (tri methyl K9) antibody ChIP Grade (Abcam, Cat# ab8898, Cambridge, MA) was used at 1:8000 and incubated overnight at 4°C. Secondary antibodies used were anti-rabbit (PV6119) or anti-mouse (PV6114) from Novocastra PowerVision Poly-HR IHC Detection System. Prostate tissue with characteristic staining patterns for H3K27me3 and H3K9me3 were used as positive/negative control, and characteristic vessel wall staining was used as an internal control for H3K27me3 staining. Once antibody concentration had been optimized with prostate tissue, tissue microarrays were stained. 5hmC detection was completed using the rabbit polyclonal 5-hmC specific antibody (Active Motif, Cat# 39769, Carlsbad, CA) at a 1:20,000 dilution for 1 hour at room temperature. The mouse monoclonal 5-methylcytosine specific antibody (Calbiochem, EMD Chemicals Inc., Cat# NA81-50UG, San Diego, CA) at a 1:2000 dilution incubated for 1 hour at room temperature was used for 5mC detection. After immunohistochemical staining, all tissue arrays were counterstained with hematoxylin.

HEK293 cells transfected with expression vectors coding for *TET2* or control vectors were used as a standard internal control for all immunolabeling experiments for 5hmC as described previously [20]. HEK293 cells show very low baseline levels of 5hmC. Overexpression of *TET2* increases 5hmC levels resulting in detectable immunoreactivity. The consistency of immunolabeling is documented in Additional file 2: Figure S1 showing 5hmC staining from 3 independent experiments. Control HEK293 cells never show immunoreactivity, whereas *TET2* transfected cells consistently show 5hmC labeling. For 5mC staining the same control slides were used.

Immunoreactivity was assessed by a neuropathologist (FJR) using H-scores (H) (0–200) which were obtained by multiplying intensity of stain (0: no stain, 1: weak stain, 2: strong stain) by percentage (0–100) of neoplastic cells showing the staining intensity [21]. Since most normal terminally differentiated cells show high 5hmC levels, all H-scores were internally normalized to the staining intensities in the non-neoplastic cells. Every case therefore served as its own internal control which greatly reduced inconsistencies due to inadequate tissue fixation.

### mRNA expression profiling

Tissue mRNA expression profiling of 6 DIPG tissue samples and adjacent normal sections [4] were reanalyzed for relative *TET* expression using Partek Genomics Suite v6.6 (Partek Incorporated, St. Louis, MO). Two of these samples are also represented in the TMA. Expression values were extracted from Partek and graphed using Prism Graph-Pad software (San Diego CA). All of the six specimens used for this study had the H3.3K27M mutation.

### Statistical analysis

Statistical analysis was completed using Graph-Pad Prism 6 software (Graph-Pad Software, La Jolla, CA). The non-parametric Kruskal-Wallis one way analysis of variance (ANOVA) test was used to identify differences between all tumor subtype’s H-score medians. The Mann–Whitney test was used to analyze differences by rank sum of H scores between two tumor subtypes.

## Results

### H3K27me3 levels are lower in DIPG compared to adult and pediatric extrapontine GBM

**Figure 1:**
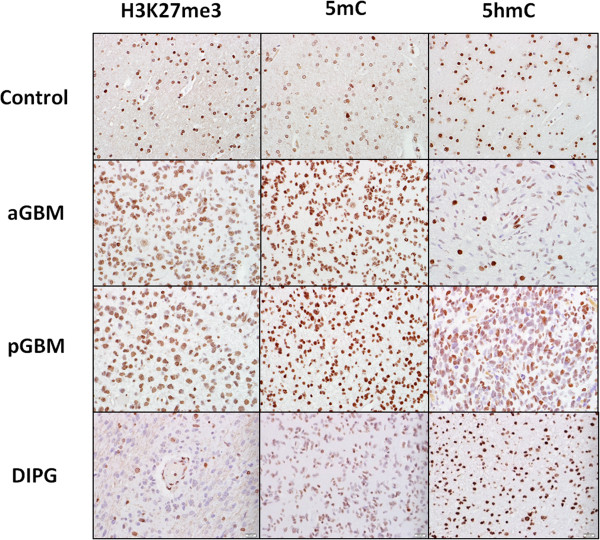
**Differential H3K27me3, 5mC and 5hmC immunoreactivity in DIPG and extrapontine GBM.** Representative micrographs (400×) of immunoreactivity for Histone 3 Lysine 27 trimethylation (H3K27me3), 5-methylcytosine (5mC), and 5-hydroxymethylcytosine (5hmC) in control brain, pediatric glioblastoma (pGBM), adult glioblastoma (aGBM), and diffuse intrinsic pontine glioma (DIPG).

**Figure 2:**
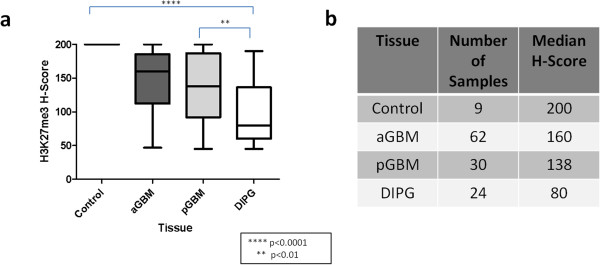
**H3K27me3 is decreased in DIPG.** H-scores for Histone 3 Lysine 27 trimethylation (H3K27me3) immunoreactivity in normal brain (Control), adult glioblastoma (aGBM), pediatric glioblastoma (pGBM), and diffuse intrinsic pontine glioma (DIPG) tissue (p < 0.0001, Kruskal-Wallis ANOVA Test) **(a)**. H3K27me3 median H-score was significantly lower for DIPGs compared to pediatric GBM (p < 0.01, Mann–Whitney Test), adult GBM (p < 0.0001), and normal brain (p < 0.0001) tissue. A significant difference was also noted in immunoreactivity between control and adult GBM (p < 0.0001) and also control and pediatric GBM (p < 0.0001). No difference was noted between adult and pediatric GBM immunoreactivity for H3K27me3 (p > 0.05). Number of samples per group and median H-scores are specified in **b**.

**Figure 3:**
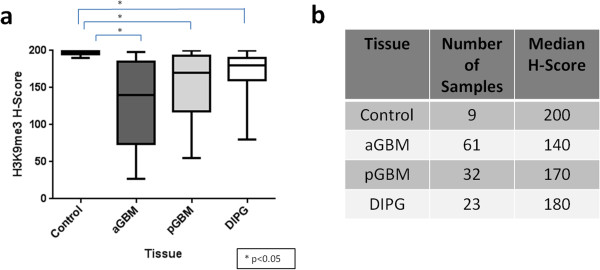
**H3K9me3 immunoreactivity is no different in DIPG, adult GBM, and pediatric GBM.** H-scores for Histone 3 Lysine 9 trimethylation (H3K9me3) immunoreactivity in normal brain (Control), adult glioblastoma (aGBM), pediatric glioblastoma (pGBM), and diffuse intrinsic pontine glioma (DIPG) tissue (p < 0.0001, Kruskal-Wallis ANOVA Test) **(a)**. H3K9me3 immunoreactivity was relatively preserved in DIPG and not significantly different from extrapontine GBM (adult GBM p > 0.05, pediatric GBM p > 0.05, Kruskal-Wallis test of multiple comparisons). A significant difference was noted between tumor groups and normal brain (p < 0.05, Kruskal-Wallis test of multiple comparisons). Number of samples per group and median H-scores are specified in **b**.

H3K27me3 nuclear immunoreactivity was present in all samples, and was particularly strong in non-neoplastic brain, including neurons, glial and endothelial cells (Figure 1). Decreased H3K27me3, defined as a H-score < 100, was present in 37/116 (32%) scorable tumors, particularly in DIPG 17/24 (71%) followed by pediatric 8/30(27%) and adult GBM 12/62(19%). H3K27me3 median H-score was significantly lower for DIPG (H = 80) compared to pediatric GBM (H = 138) (p <0.01, Mann–Whitney Test), adult GBM (H = 160) (p < 0.0001), and normal brain (H-score 200) (p < 0.0001) tissue (Figure 2). A significant difference was also noted in immunoreactivity between control and adult GBM (p < 0.0001) and also control and pediatric GBM (p < 0.0001). No difference was noted between adult and pediatric GBM immunoreactivity for H3K27me3 (p > 0.05). Conversely, H3K9me3 immunoreactivity was relatively preserved in DIPG (H = 180) and not significantly different from extrapontine GBM (adult GBM H = 140, pediatric GBM H = 170, p > 0.05, Kruskal-Wallis test of multiple comparisons). A significant difference was noted between tumor groups and normal brain (H = 200, p < 0.05, Kruskal-Wallis test of multiple comparisons) explaining the Kruskal-Wallis ANOVA p < 0.0001 (Figure 3).

### DIPGs have increased 5hmC and decreased 5mC compared with extrapontine GBM

**Figure 4:**
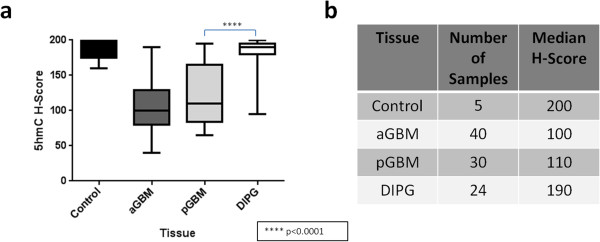
**5hmC is increased in DIPG.** H-scores for 5-hydroxymethylcytosine (5hmC) immunoreactivity in normal brain (control), adult glioblastoma (aGBM), pediatric glioblastoma (pGBM), and diffuse intrinsic pontine glioma (DIPG) tissue (p < 0.0001, Kruskal-Wallis ANOVA Test) **(a)**. Number of samples per group and median H-scores are specified in **b**.

**Figure 5:**
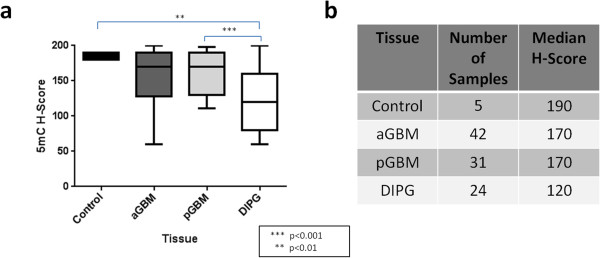
**5mC is decreased in DIPG.** H-scores for 5-methylcytosine (5mC) immunoreactivity in normal brain (Control), adult glioblastoma (aGBM), pediatric glioblastoma (pGBM), and diffuse intrinsic pontine glioma (DIPG) tissue (p < 0.0024, Kruskal-Wallis ANOVA Test). **(a)**. Number of samples per group and median H-scores are specified in **b**.

Because alterations in DNA methylation levels represent an important feature of DIPG, we next studied 5mC and 5hmC levels in a total of 94 scorable tumors from 3 tissue microarrays (Figure 1). 5hmC immunoreactivity was significantly higher for DIPG (H = 190) compared to pediatric GBM (H = 110) (p < 0.0001) and adult GBM (H = 100) (p < 0.0001). No significant difference in H-scores was noted between DIPG tissue and normal brain (H = 200) (p = 0.23). There was no significant difference in 5hmC H-score between adult and pediatric GBM (p = 0.18) tissue (Figure 4). Conversely, 5mC was significantly lower in DIPGs (H = 120) compared to pediatric GBM (H = 170) (p < 0.001), adult GBM (H = 170) (p < 0.01), and normal brain (H = 190) (p < 0.01). No significant difference in H-scores was noted between adult and pediatric GBM (p = 0.61) tissue for 5mC (Figure 5).

### DIPGs have increased mRNA expression of *TET1* and *TET3* relative to normal brain

**Figure 6:**
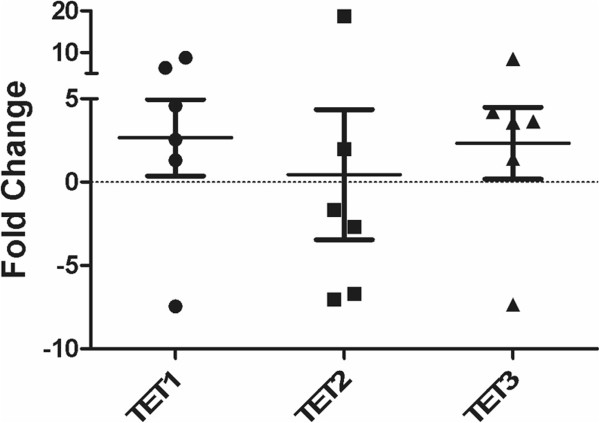
***TET1*** **and** ***TET3*** **expression are increased in DIPG tumors relative to normal brain.** mRNA expression profiling of *H3F3A* mutant DIPG tumor tissue shows relative increased expression of *TET1* and *TET3*, but not *TET2*, in tumor relative to adjacent non-neoplastic brain control.

Comparative analysis of DIPG tumor tissue and non-neoplastic brain sections showed increased *TET1* and *TET3* mRNA expression in tumor relative to patient matched brain (as expressed in fold change). *TET2* expression was not increased relative to normal brain (Figure 6).

## Discussion

We determined by IHC relative levels of H3K27me3, 5mC, and 5hmC in DIPG, pediatric GBM, and adult GBM tissue. Our data are consistent with previously reported low levels of H3K27me3 due to the H3K27M mutation in the majority of DIPG. H3K9me3 in DIPG was not significantly different from H3K9me3 immunoreactivity in extrapontine GBM and unlikely to be playing a role in the tumorigenicity of DIPG. Surprisingly, 5hmC immunoreactivity was significantly elevated in DIPG, in contrast to 5mC, suggesting that imbalance between 5hmC and 5mC plays a global role in the biology of DIPG. In general, high levels of 5hmC have been shown to be a feature of terminally differentiated cells [20]. In numerous solid tumors including carcinoma of the breast, prostate, colon, melanoma, and gliomas, 5hmC levels appear to be greatly reduced in neoplastic cells [20]. Our group has previously shown that in adult GBM and anaplastic astrocytoma high levels of 5hmC are associated with a less aggressive phenotype [21]. However, the global DNA hypomethylation that occurs in DIPG in conjunction with relatively increased 5hmC may represent signs of a novel global epigenetic dysregulation state distinct from that of adult high grade glioma. The elevation of 5hmC in DIPG must be considered in the context of global loss of 5mC in these tumors. The striking imbalance between 5hmC and 5mC in DIPG may be another sign of the marked epigenetic dysregulation which underlies these aggressive tumors.

A limitation of our study is the relative lack of clinical data available for the patient samples. We do not know which, if any, chemotherapeutic agents patients have been treated with and whether they have received radiation. Our findings may be affected by prior treatment but the internal consistency of our findings within tumor groups supports their significance. The underlying cause for the shift in balance towards increased 5hmC expression relative to 5mC in DIPG compared to normal brain and extrapontine GBM is unclear. However, one possibility is altered activity of TETs. Preliminary evaluation of *TET* mRNA expression shows elevation of *TET1* and *TET3* in DIPG compared to control brain. In other cancer types, *TET* is mutated and inactivated, leading to globally decreased 5hmC and increased DNA methylation [17]. In contrast, a possible result of the loss of global DNA methylation could be deposition of the 5hmC intermediary and epigenetic imbalance.

**Figure 7:**
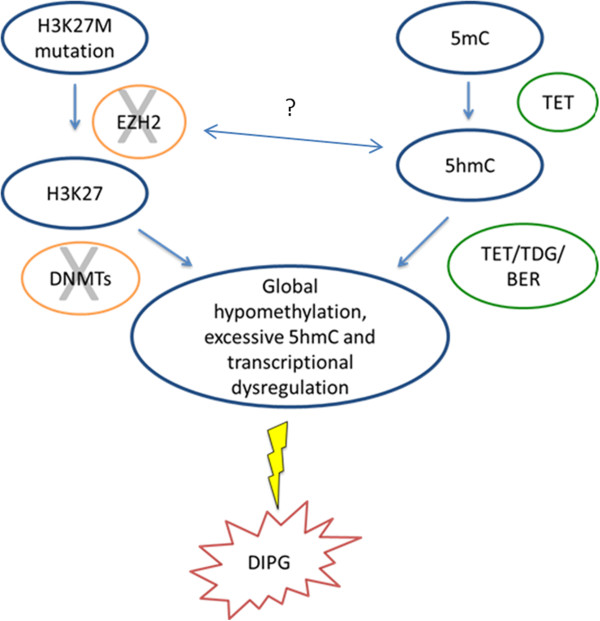
**Overproduction of 5hmC and disruption of histone methylation may contribute to DIPG tumorigenesis.** Normal neural development is controlled by histone and DNA methylation executed by enhancer of zeste homologue 2 (EZH2) methyltransferase, DNA methyltransferase (DNMT), Ten Eleven Translocation (TET), Thymine-DNA glycosylase (TDG), and base excision repair (BER). Dysregulation of the epigenome by *H3F3A* mutation/EZH2 inhibition and corresponding overproduction of 5hmC by TET in DIPG may promote DIPG tumorigenicity.

During differentiation, global levels of 5hmC and H3K27me3 are tightly co-regulated and in most solid tumors both marks are concordantly reduced [24]. The data presented here suggests that DIPGs have a unique epigenetic fingerprint featuring high 5hmC and loss of H3K27me3. This epigenetic constellation may contribute to DIPG tumor initiation and maintenance. The alteration in H3.3 which inactivates EZH2 and global DNA hypomethylation suggests that two regulatory pathways that communicate to maintain normal brain development are altered in this tumor type, leading to an unopposed active transcriptional state (Figure 7). Elevated 5hmC and depressed H3K27me3 in normal brain development are associated with neural differentiation and affect neural migration through inhibition of EZH2 (part of the PRC2 complex) [20, 18]. We hypothesize that migration is aberrant in DIPG since EZH2 is no longer able to function at Lysine 27 in Histone 3.3. Elevated 5hmC is found in differentiating cells and usually in conjunction with H3K27me3 loss. There is a possibility that the Histone 3 and cytosine methylation pathways cross-talk and co-regulate each other. However, in DIPG, aberrant TET enzyme expression may result in loss of 5mC and increased 5hmC. This may lead to aberrant PRC2 sequestration from primary targets, a transcriptionally active state, and tumorigenicity.

Histone and cytosine methylation dysregulation is unique to DIPG. When neither pathway is able to function in its regulatory role in neural development, DIPG develops. This epigenetic imbalance may underlie DIPG tumor formation and resistance to treatment. Our work supports recent findings indicating that H3K27me3 loss, through the dominant negative H3K27M mutation, is associated with DNA hypomethylation and an increase in transcriptionally upregulated genes. This suggests that there is crosstalk between histone methylation pathways and DNA methylation pathways leading to changes in transcriptional activity [10].

## Conclusions

The H3.3 Lysine 27 is a critical site for the deposition of an inhibitory epigenetic mark and is mutated in the majority of DIPG, suggesting that targeting epigenetics could be one therapeutic approach for this highly aggressive pediatric tumor. Understanding the epigenetic landscape of DIPG opens up the opportunity for epigenetic modifiers, which could potentially shift the active genome of this deadly tumor into a silent and regulated state. The role of epigenetic modifiers such as methylation inhibitors and histone deacetylase inhibitors [7] in treating DIPG needs to be reevaluated as the epigenetic mechanisms of DIPG are better understood.

## Electronic supplementary material

Additional file 1: Table S1: DIPG specimen demographic and histological characteristics adapted from Ballester et al. (-) data not available. (DOCX 14 KB)

Additional file 2: Figure S1: HEK293 cells are positive controls for 5mC IHC and negative controls for 5hmC. *TET* overexpressed HEK293 cells function as positive controls for 5hmC IHC. Figure 1a is a representative image of IHC staining of HEK293 cells for 5mC (positive control). Figure 1b shows representative images from three different rounds of staining for 5hmC with HEK293 cells overexpressed for *TET2* stained for 5hmC on the right (positive control) and control transfected HEK293 cells not showing any immunoreactivity for 5hmC on the left (negative control). (PPTX 464 KB)

## Abbreviations

5hmC: 5-hydroxymethylcytosine
5mC: 5-methylcytosine
aGBM: Adult glioblastoma
α-KG: Alpha-ketoglutarate
BER: Base excision repair
DIPG: Diffuse intrinsic pontine glioma
DNMT: DNA methyltransferase
EZH2: Enhancer of zeste homologue 2
GBM: Glioblastoma
H: H-Score
H3K9me3: Histone 3 Lysine 9 trimethylation
H3K27: Histone 3 Lysine at position 27
H3K27me3: Histone 3 Lysine 27 trimethylation
K27M: Lysine at position 27 of histone 3 changed to methionine
PcG: Polycomb group
pGBM: Pediatric glioblastoma
PRC2: Polycomb repressive complex 2
SHH: Hedgehog
TET: Ten eEleven translocation
TDG: Thymine DNA glycosylase.
