# Supplementary material for: Increased 5-hydroxymethylcytosine and decreased 5-methylcytosine are indicators of global epigenetic dysregulation in diffuse intrinsic pontine glioma
Source: Acta Neuropathol Commun. 2014 Jun 3;2:59. doi: 10.1186/2051-5960-2-59 (PMC4229804; doi:10.1186/2051-5960-2-59)
Supplement: Supplementary file 1 — Additional file 1: Table S1: DIPG specimen demographic and histological characteristics adapted from Ballester et al. (-) data not available. (DOCX 14 KB) [file 40478_2014_130_MOESM1_ESM.docx]

**Supplementary Table 1:** DIPG specimen demographic and histological characteristics adapted from Ballester et al. (-) data not available.

| **Case** | **Age** | **Sex** | **Histological Grade** |
| --- | --- | --- | --- |
| 1 | 8 | M | IV |
| 2 | 7 | M | IV |
| 3 | 14 | F | IV |
| 4 | 11 | M | IV |
| 5 | 15 | F | II |
| 6 | 8 | M | IV |
| 7 | 6 | M | IV |
| 8 | 5 | F | IV |
| 9 | 13 | F | IV |
| 10 | 6 | M | IV |
| 11 | 9 | F | IV |
| 12 | - | - | IV |
| 13 | 3 | F | III |
| 14 | - | - | IV |
| 15 | - | - | IV |
| 16 | - | - | II-III |
| 17 | 3 | M | IV |
| 18 | - | M | III |
| 19 | 7 | M | IV |
| 20 | 4 | F | III |
| 21 | 6 | F | IV |
| 22 | 5 | M | IV |
| 23 | 13 | M | III |
| 24 | 8 | F | IV |
