# Supplementary figures and images for: Increased 5-hydroxymethylcytosine and decreased 5-methylcytosine are indicators of global epigenetic dysregulation in diffuse intrinsic pontine glioma
Source: Acta Neuropathol Commun. 2014 Jun 3;2:59. doi: 10.1186/2051-5960-2-59 (PMC4229804; doi:10.1186/2051-5960-2-59)

## Slide 1
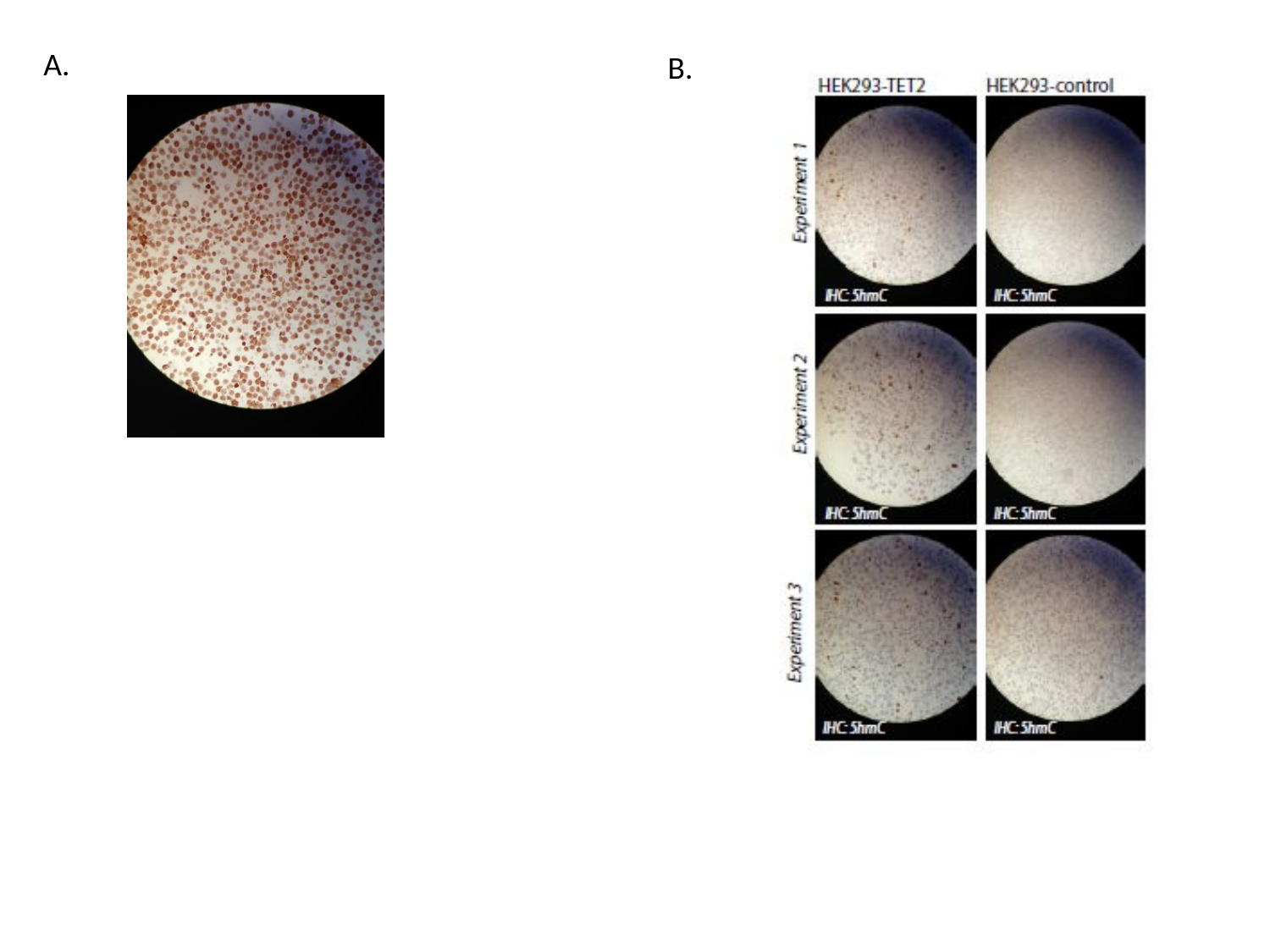

A.
B.

Supplement: Supplementary file 2 — Additional file 2: Figure S1: HEK293 cells are positive controls for 5mC IHC and negative controls for 5hmC. TET overexpressed HEK293 cells function as positive controls for 5hmC IHC. Figure 1a is a representative image of IHC staining of HEK293 cells for 5mC (positive control). Figure 1b shows representative images from three different rounds of staining for 5hmC with HEK293 cells overexpressed for TET2 stained for 5hmC on the right (positive control) and control transfected HEK293 cells not showing any immunoreactivity for 5hmC on the left (negative control). (PPTX 464 KB) [file 40478_2014_130_MOESM2_ESM.pptx]
